# Mesenchymal Stromal Cells in Neuroblastoma: Exploring Crosstalk and Therapeutic Implications

**DOI:** 10.1089/scd.2020.0142

**Published:** 2021-01-15

**Authors:** Caroline Hochheuser, Laurens J. Windt, Nina Y. Kunze, Dieuwke L. de Vos, Godelieve A.M. Tytgat, Carlijn Voermans, Ilse Timmerman

**Affiliations:** ^1^ Sanquin Research and Landsteiner Laboratory, Department of Hematopoiesis, Amsterdam UMC, University of Amsterdam, Amsterdam, the Netherlands.; ^2^ Princess Maxima Center for Pediatric Oncology, Utrecht, the Netherlands.

**Keywords:** neuroblastoma, mesenchymal stromal cells, metastasis, bone marrow, chemoresistance, targeted therapy

## Abstract

Neuroblastoma (NB) is the second most common solid cancer in childhood, accounting for 15% of cancer-related deaths in children. In high-risk NB patients, the majority suffers from metastasis. Despite intensive multimodal treatment, long-term survival remains <40%. The bone marrow (BM) is among the most common sites of distant metastasis in patients with high-risk NB. In this environment, small populations of tumor cells can persist after treatment (minimal residual disease) and induce relapse. Therapy resistance of these residual tumor cells in BM remains a major obstacle for the cure of NB. A detailed understanding of the microenvironment and its role in tumor progression is of utmost importance for improving the treatment efficiency of NB. In BM, mesenchymal stromal cells (MSCs) constitute an important part of the microenvironment, where they support hematopoiesis and modulate immune responses. Their role in tumor progression is not completely understood, especially for NB. Although MSCs have been found to promote epithelial–mesenchymal transition, tumor growth, and metastasis and to induce chemoresistance, some reports point toward a tumor-suppressive effect of MSCs. In this review, we aim to compile current knowledge about the role of MSCs in NB development and progression. We evaluate arguments that depict tumor-supportive versus -suppressive properties of MSCs in the context of NB and give an overview of factors involved in MSC-NB crosstalk. A focus lies on the BM as a metastatic niche, since that is the predominant site for NB metastasis and relapse. Finally, we will present opportunities and challenges for therapeutic targeting of MSCs in the BM microenvironment.

## Introduction

Constituting 7%–10% of all childhood malignancies, neuroblastoma (NB) is the second most common solid childhood tumor [1,2]. The tumors arise from neuroepithelial cells that migrate from the neural crest to form the sympathetic nervous system in embryonic development [3]. This origin explains some of the most prominent features of the disease: both localization and genetic features are highly heterogeneous, with primary tumors located in various locations of the sympathetic nervous system, most frequently in the adrenal medulla and paraspinal ganglia. Furthermore, similar to sympathetic neurons, NB tumors secrete catecholamines [4,5].

At the time of diagnosis, about 50% of the patients present with disseminated disease [6]. With an incidence rate of >90% in high-risk patients, the bone marrow (BM) is the most frequent site of metastasis [7,8]. To tailor treatment according to the severity of disease, an International Neuroblastoma Risk Group (INRG) classification system has been established and updated throughout the years [9]. Today, patients are classified into very low-, low-, intermediate-, and high-risk groups. Key factors that classify patients into the high-risk group are dissemination status, age >18 months at diagnosis, MYCN amplification, rearrangements of the TERT locus, inactivating mutations in ATRX and chromosome 11q aberration [10–12].

Although nonhigh-risk groups have an excellent prognosis with survival rates of >90% without intensive treatment, the standard-of-care treatment strategy for high-risk patients is much more complex. It includes induction therapy, surgical resection of the primary tumor, high-dose myeloablative chemotherapy with autologous hematopoietic stem cell (HSC) transplantation, radiation therapy, and postconsolidation immunotherapy consisting of antidisialoganglioside (GD2)- and isotretinoin treatment [13]. Despite this intense treatment, >30% of high-risk patients experience relapse [1] and their 5-year overall survival rate remains <40% [14].

Relapse mainly emerges from those tumor cells that survive therapy and remain undetected [minimal residual disease (MRD)]. In the context of various cancer types, these residual cells have been described to adopt a nonproliferative and highly chemoresistant dormant state [15,16]. The cellular and molecular foundation of dormancy, however, as well as its role in NB metastasis are poorly understood. Interestingly, similar to the quiescence of HSCs, the BM might provide favorable conditions for the development of tumor cell dormancy [17].

## The Microenvironment in the BM

The BM is the primary site of hematopoiesis and comprises a multitude of cell types, mainly of the hematopoietic and mesenchymal lineage. The hematopoietic stem and progenitor cells (HSPCs) found in these niches, giving rise to immune cells and osteoclasts, maintain a balance of self-renewal and differentiation, which is regulated primarily by signals from the stromal microenvironment [18]. The term “stroma” comprises all nonhematopoietic cells, ie., cells of the mesenchymal lineage, deriving from mesenchymal stromal cells (MSCs), endothelial cells, and nerve cells. Among the BM stromal cell types that are relevant within the tumor microenvironment (TME) are MSCs and their descendants (adipocytes and osteoblasts), fibroblasts and endothelial cells (recently reviewed by Shiozawa [19]). This review focuses on the role of MSCs within the TME.

In the past the acronym MSC has been used for “mesenchymal stem cells,” but is nowadays used in a wider context to include cells whose biologic characteristics do not meet the definition of stem cells [20]. In this review, we use the term MSC to describe multipotent mesenchymal stromal cells. The latter are characterized in vitro by the International Society for Cellular Therapy (ISCT) as cells that (i) express CD105, CD73, and CD90, and lack expression of CD45, CD34, CD14 or CD11b, CD79a, or CD19, and HLA-DR surface molecules, (ii) have the potential to differentiate into osteoblasts, adipocytes, and chondroblasts, and (iii) adhere to plastic in standard culture conditions [21].

In the human body they can be found in various organs and tissues, including the umbilical cord, adipose tissue, placenta, and dental pulp. In fact, MSCs have been described to be present in nearly all postnatal organs and vascularized tissues [22,23]. Within the BM, their main functions are hematopoietic support, immunomodulation, and bone remodeling, which they achieve through physical contact and secretion of soluble factors [24–27].

Important to note when interpreting data from MSC studies is that essential differences exist between primary MSCs directly derived from human BM (BM-MSCs) and (i) culture-expanded MSCs, (ii) MSCs from other human tissues, and (iii) MSCs from other species, for example mouse. (i) Cultured MSCs do not perfectly reflect the properties and physiological functions of MSCs in vivo as they are known to alter the expression of cell surface markers such as CD146, CD271, CD106, and CD44 (I. Timmerman, personal observation, [28–30]) and to impair their capacity for BM-homing [31], hematopoietic support [30], and multilineage differentiation [29]. (ii) MSCs from various human tissues differ from BM-MSCs in their expression of cell surface markers (Rojewski et al. [32] compiled a comprehensive summary of marker expression on MSCs from various tissues), and furthermore in their protein expression profile, and differentiation potency [33,34]. (iii) Characterization of MSCs in other species and translating findings to the human setting is difficult due to the heterogeneity of surface markers expressed in each species (comprehensively reviewed by Boxall and Jones [35]). Mouse models are especially frequently used for in vivo studies of MSCs in the BM niche. Various markers are shared by human and mouse MSCs (eg, CD105, CD73, CD51, platelet-derived growth factor receptor alpha and beta [PDGFRα,β/CD140a,b] [36]), whereas others are predominantly studied in mouse models (Nestin [37], neuron-glial antigen 2 [NG2] [38], Leptin receptor [LepR] [39]). Although the latter have also been shown to be expressed in human MSCs [28,40–42], the concrete function of these cells in the human BM, especially in the metastatic setting of NB, has not yet been addressed.

Overall, insight obtained from studies with mouse MSCs cannot necessarily translate to the human context and require further validation. An interesting approach for avoiding these interspecies differences and studying a human-like environment in a mouse model is the xenotransplantation of a “humanized bone-marrow-ossicle niche,” derived from BM-MSCs [43].

The experimental details and important findings of key studies investigating MSCs in the NB context are summarized in Table 1 to facilitate comprehensive understanding of the studies' content.

**Table 1.** Overview of Important Findings and Experimental Procedures of Key Studies Investigating the Contribution of Mesenchymal Stromal Cells to Neuroblastoma

Topic	Key findings	Component involved	Mesenchymal stromal cells	Tumor cells	Experimental system	Reference
Tumor-suppressive	MSCs reduced primary tumor growth and prolonged survival of mice, decreased proliferation, and increased apoptosis of tumor cells (ex vivo)	*Caspase-3*	hBM-MSCs^*^ from healthy donors	hNB cell line ACN	In vivo (mouse), ex vivo (FFPE tumor specimens)	Bianchi et al. [68]
Tumor-supportive						
MSC homing to tumor site	i.p.-injected MSCs migrated to tumor (i.v.-injected MSCs did not)	—	hAT-MSC^*^ from healthy donors	None (TH-MYCN transgenic mice)	In vivo (mouse), ex vivo (FFPE tumor specimens)	Kimura et al. [51]
	MSCs given to responders expressed higher CXCR1, CCR1, (CXCR4) levels/MSCs migrated toward CCL5, CXCL12 and tumor cells	**CXCR1**, **CCR1** (CCL5), **CXCR4** (CXCL12)	hBM-MSCs^*^ from NB patients	hNB cell line NB1691	In vivo (clinical trial), in vitro	Melen et al. [52]
General tumor-supportive effects	Increased tumor cell proliferation and survival in vitro, tumor growth in vivo	**IL6**, **IL8**, **CCL2**, **CXCL12**, *JAK2/STAT3 MEK/ERK1,2*	Primary CAF-like MSCs and BM-MSCs from NB patients	hNB cell lines CHLA-255, SK-N-SH, SK-N-BE2, CHLA-90	In vitro, ex vivo (FFPE tumor specimens), in vivo (mouse)	Borriello et al. [73]
	Secretion of protumorigenic cytokines and chemokines from BM-MSCs	*Exosomes*, **IL6**, **IL8**, **VEGF**, **CCL2**, **ERK1/2**	hBM-MSCs from NB patients	9 hNB cell lines	In vitro	Nakata et al. [129]
	Increased proliferation of NB cells, tumor growth in vivo, increased IL6 in serum and BM of patients	**IL6**, *STAT3/ERK*	hBM stromal cells	11 hNB cell lines	In vitro, ex vivo (patient serum samples), in vivo (mouse)	Ara et al. [135]
	Gal3BP induced IL6 secretion from BM stromal cells	*Gal3BP*, **IL6**, **ERK1/2**	hBM stromal cells	hNB cell lines CHLA-255, SK-N-BE(2), NB19	In vitro	Fukaya et al. [133]
	Transcriptional upregulation of IL-6 in BM-MSC, Gal3BP present in tumor cells and ECM of 96% of tumor specimen	**Gal-3BP/Ras/MEK/ERK signaling**, *Gal3BP*	hBM-MSCs	9 hNB cell lines	In vitro, ex vivo (FFPE tumor specimens)	Silverman et al. [134]
Stimulation of metastasis, BM invasion	BM-MSC secretome promoted invasiveness in 4 of 5 cell lines studied	*CXCR4*, *MMP-9*	hBM-MSC-TERT	In total 20 hNB cell lines (5 for invasion assay)	In vitro	Shankar et al. [100]
	MSC secretome increased migration and invasiveness of NB cells	**CXCL12**, *CXCR4*, *CXCR7*	hBM-MSCs, hBM-MSC-TERT	hNB cell lines BE(2)-M17, BE(2)-C, IMR32, SK-N-LP, SH-SY5Y	In vitro	Ma et al. [109]
	MSC secretome enhanced migratory capacity in 2 of 3 cell lines	*CXCR4*, CXCL12	hBM-MSCs^*^ from healthy donors	hNB cell lines SH-SY5Y, GI-LI-N and Htla-230	In vitro	Bianchi et al. [68]
Chemoresistance/dormancy	Protection from etoposide-induced apoptosis	**IL6**, *STAT3*	hBM-MSCs from healthy donors	hNB cell lines	In vitro, ex vivo (FFPE tumor specimens)	Ara et al. [180]
	hMSCs and monocytes impair anti-NB activity of aNK/anti-GD2-immunotherapy	TGF-β1	hBM-MSCs from NB patients	hNB cell lines CHLA-255, CHLA-136	In vitro, in vivo (mouse)	Wu et al. [182]
	protection from etoposide-induced apoptosis	*S1PR1*, *JAK-STAT3 signaling*	hBM-MSCs from NB patients	hNB cell lines CHLA-171, CHLA-255	In vitro, in vivo (mouse)	Lifshitz et al. [179]
Altered bone homeostasis	BM-MSCs drove bone lesions through osteoclast activation	**IL6**	hBM-MSCs^*^ from healthy donors	hNB line CHLA-255, rat osteoclasts	In vitro	Sohara et al. [151]
	Increased osteogenic differentiation of MSCs	BMP4, **VEGFa**	Primary murine BM-MSCs	hNB cell lines CHLA-255 and SK-N-BE	In vitro	HaDuong et al. [153]
	Increased number of MSCs, osteogenic differentiation of MSCs, presence of a MSC subtype	—	hBM-MSCs from NB patients	—	In vitro, ex vivo (BM biopsies)	Hochheuser et al. [113]
	NB cells decreased osteogenic differentiation capacity of MSCs	*Dkk1*	hMSCs^*^ from healthy pediatric donors	hNB cell lines SH-SY5Y, LAN1, CHP212, NB100	In vitro	Granchi et al. [161]

*Italic*, cancer-derived.

*Bold*, MSC-derived.

aNK, activated human natural killer cells; BM, bone marrow; CAF, cancer-associated fibroblast; CCL5, CC chemokine ligand 5; CCR1, CC chemokine receptor 1; CXCR1, C-X-C motif chemokine receptor-1; Dkk1, Dickkopf-related protein-1; ECM, extracellular matrix; FFPE, formalin-fixed paraffin-embedded; Gal-3BP, Galectin-3 binding protein; hAT, human adipose tissue-derived; hBM, human bone marrow-derived; hBM-MSC-TERT, human MSCs immortalized by enforcing the expression of TERT in primary bone marrow MSCs; hNB, human neuroblastoma; i.p., intraperitoneally; i.v., intravenously; MMP-9, matrix metalloproteinase-9; MSC, mesenchymal stromal cell; MSC^*^, mesenchymal *stem* cells; NB, neuroblastoma; TGF-β, transforming growth factor-β; VEGF, vascular endothelial growth factor.

## Contribution of MSCs to NB Development and Progression

**FIG. 1.**
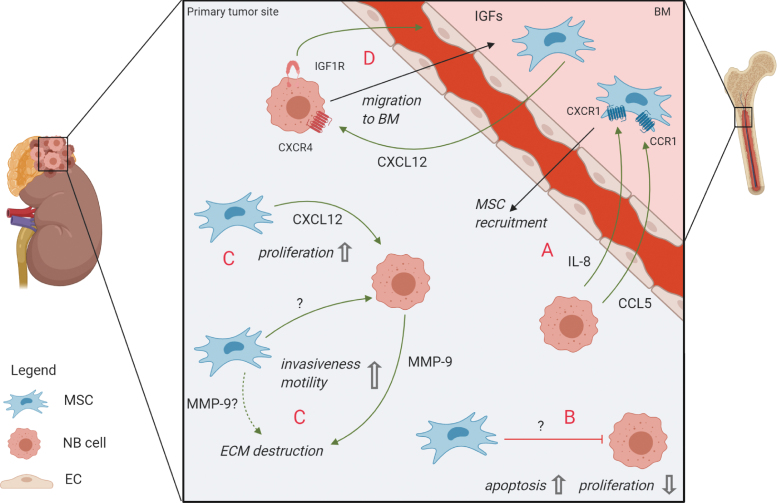
Crosstalk between MSCs and NB cells at the primary tumor site and migration to/from the BM. **(A)** MSCs are attracted from the BM to the primary site (among others through CXCR1/IL-8 and CCR1/CCL5 signaling) [52]. **(B)** Unknown MSC-derived mediators can exert a tumor-suppressive effect [68]. **(C)** The CXCR4/CXCL12 axis plays a role in proliferation and survival of tumor cells and decreased apoptosis rates [74]. MMP-9 [99,100] might play a role in promoting EMT and metastasis: unknown signaling events from MSCs induce MMP-9 expression in NB cells [100], whereas MSCs potentially also secrete MMP-9 themselves (*dashed line*). **(D)** NB cells are attracted to the BM metastatic niche through the CXCR4/CXCL12 axis [100,109] and can dock to the BM endothelial cells (ECs) through IGF-1R, subsequently migrating toward IGF-1 in the BM stroma [115]. BM, bone marrow; CCR1/CCL5, CC chemokine receptor 1/CC chemokine ligand 5; CXCR1, C-X-C motif chemokine receptor-1; ECM, extracellular matrix; EMT, epithelial-to-mesenchymal transition; IGF-1, insulin-like growth factor 1; IL-8, interleukin-8; MMP-9, matrix metalloproteinase-9; MSC, mesenchymal stromal cell; NB, neuroblastoma. Color images are available online.

Various forms of interaction between NB cells and the TME at the primary tumor site have been described (Fig. 1). The inflammatory environment of tumors is known to recruit MSCs to the TME in many cancer types [44,45]. Numerous signaling molecules, including stromal derived factor-1 (SDF-1/CXCL12), transforming growth factor-β (TGF-β), interleukin-8 (IL-8), matrix metalloproteinase-1 (MMP-1), and monocyte chemoattractant protein 1 (MCP-1/CCL2) were shown to be involved in MSC recruitment to the primary tumor site [46–49]. A detailed overview of MSC migration to tumors and healthy organs, including chemotactic stimuli, is given by Cornelissen et al. [50].

In NB, adipose tissue-derived MSCs were demonstrated to successfully migrate to primary NB tumors in mice when injected intraperitoneally [51]. An in vitro evaluation of a clinical trial for oncolytic virotherapy with 12 patients revealed that receptor/ligand pairs C-X-C motif chemokine receptor-1 (CXCR1)/IL-8 and CC chemokine receptor 1/CC chemokine ligand 5 (CCR1/CCL5) were involved in successful migration of MSCs to the tumor [52] (Fig. 1A).

Once MSCs are part of the microenvironment, they directly or indirectly interact with tumor cells [53]. These interactions can either have phenotypic and functional effects on MSCs themselves, or induce signaling from MSCs to other cell types in the stroma through chemokines or extracellular vesicles (EVs) [54–56]. Both supportive and inhibitory effects on the tumor resulting from these interactions have been described, depending on the cancer type, localization of the tumor, investigation method (in vitro vs. in vivo), and number and origin of MSCs [57].

### MSCs exhibiting tumor-suppressive effects

Early evidence of tumor-suppressive effects by the tumor stroma originates from studies from the 1990s and 2000s before a clear concept of MSCs had been developed: “(adherent) BM stromal cells” were described to inhibit the growth of leukemia [58], lung carcinoma [59], and colon carcinoma [60]. Later, MSCs have been demonstrated to inhibit glioma cell proliferation in vitro [61] and to have inhibitory effects on the in vivo growth and metastasis of Kaposi-sarcoma [62], breast cancer [63], and various hematological malignancies (reviewed extensively by Lee et al. [64]).

Some mechanistic insights into the tumor-suppressive effect of MSCs implicate a role of Wnt signaling [65]. Both *activation* of (noncanonical) Wnt signaling by MSC-derived Wnt5a as well as *inhibition* of (canonical) Wnt-signaling by MSC-derived Dickkopf-related protein-1 (Dkk1) have been shown to decrease proliferation rates in two leukemia cell lines [66,67]. Concrete mechanistic evidence for tumor-suppressive functions of MSCs in NB is sparse. One study revealed that intratumoral injection of MSCs into primary NB tumors in mice significantly reduced tumor growth and prolonged survival of tumor-bearing mice. These effects were mediated by decreased proliferation and higher apoptosis rates of tumor cells [68] (Fig. 1B). However, assessment of proliferation in an in vitro setting within the same study revealed that MSCs could not only inhibit but also promote proliferation of NB cells, depending on the cell line used. The effect of MSCs on NB tumors is, therefore, not clearly defined and is instead—in this context—dependent on the NB cell line used.

### MSCs exhibiting tumor-supportive effects

In contrast to these tumor-suppressive effects of MSCs, multiple studies describe a tumor-supportive role of MSCs instead. Studies in breast cancer (in vitro and in vivo*)* [69], prostate cancer (PC; in vitro) [70], adenocarcinoma and Lewis lung carcinoma (in vitro and in vivo) [71] demonstrated a beneficial effect of MSCs on tumor growth, cell survival, drug resistance, and angiogenesis. According to studies on several tumor types, it is believed that upon arrival at the primary tumor site, BM-MSCs adapt a cancer-associated fibroblast (CAF)-like phenotype, while still retaining surface marker expression and differentiation potential that is characteristic for MSCs [49,72,73]. In NB, it was shown that these CAF-like MSCs as well as normal BM-MSCs enhance tumor cell proliferation and survival in vitro and stimulate tumor engraftment and growth in vivo through the JAK2/STAT3 and MEK/ERK1/2 pathways in NB cells [73]. The connection between MSCs and CAFs is described in more detail in Box 1.

Furthermore, the CXCL12/CXCR4 axis has been implicated in local tumor-supporting effects: experiments with NB cell lines and an orthotopic NB mouse model revealed a CXCL12-dependent beneficial effect of CXCR4 on tumor growth and -survival [74] (Fig. 1C). In the healthy BM setting, expression of CXCL12 in human and murine MSCs has been shown, for example, in studies by Kortesidis et al. [75] and Méndez-Ferrer et al. [76], who had characterized MSCs by expression of Stro1 and Nestin, respectively, as well as their clonogenicity and trilineage differentiation potential. An additional source of CXCL12 in the BM is likely to be constituted by MSC's progeny like osteoblasts and/or other stromal cells like endothelial and perivascular cells [25,77–79]. Interestingly, in a recent study our group has also detected CXCL12 expression in primary MSCs from metastatic BM samples of NB patients (I. Timmerman, C. Hochheuser, personal observation). Other prominent functions of CXCL12/CXCR4 signaling regarding metastasis are discussed below.

**Box 1. MSCs and CAFs**

MSCs were first associated with CAFs after BM-derived myofibroblasts were reported to accumulate in tumor stroma and to constitute up to 25% of stromal fibroblasts [80–83]. Subsequently, the question arose whether MSCs differentiate into CAFs or only share certain characteristics with CAFs. It is, therefore, important to define this term: CAFs are cells in the TME defined by (a subset of) the following characteristics: increased proliferation and migration, a “CAF gene expression signature,” activation of TGF-β-, mitogen-activated protein kinase (MAPK)- and nuclear factor kappa-light-chain-enhancer of activated B cells (NF-κB) signaling, and expression of for example α-fibroblast activation protein (αFAP), fibroblast-specific protein-1 (FSP-1), and alpha-smooth muscle actin (α-SMA) [72,73,84–87]. A definition based on genomic landscape, distinct surface markers or cell of origin, however, is lacking.

Madar et al. [88] suggested to define CAF “as a ‘state’ rather than a cell type,” meaning that several different cell types, such as MSCs, fibroblasts, epithelial cells, and tumor cells that have undergone EMT can adapt CAF traits (ie, mesenchymal appearance and tumor-supportive effects). This perception is in line with the finding that (only) up to 20% of CAFs derive from MSCs, implying that the other 80% must derive from other sources [89]. CAF is, therefore, merely to be understood as a “label” that a cell gets once it becomes part of the TME and supports tumorigenesis.

## Stimulation of Metastasis

MSCs do not only exert a local tumor-supportive effect at the primary tumor site, but also contribute to metastasis of tumor cells. Two major processes leading to metastasis are EMT, which allows tumor cells to detach from the primary tumor site, and subsequent metastatic migration to distant sites facilitated by adhesion molecules [90,91].

### Epithelial-to-mesenchymal transition

During EMT, tumor cells undergo a change in cellular structure and expression of surface molecules until their morphological phenotype resembles that of mesenchymal rather than epithelial cells [91]. Interestingly, this event also happens during embryonic development of the sympathetic nervous system as neuroepithelial cells detach from the neural crest. Researchers, therefore, propose that in special cases of NB, a natural BM dissemination can originate from an early mutation event during the migration of neural crest cells [92].

Although a few factors involved in NB EMT have been discovered [93–95], it is poorly understood to what extent MSCs promote this process. TGF-β, for example, has been described to cause functional changes in NB cells that are characteristic for EMT: upon treatment with recombinant human TGF-β1, NB cells showed a lower expression of adhesion molecule and epithelial marker E-cadherin, a higher expression of fibroblast marker a-SMA, and were generally more motile [93]. MSCs from healthy adult BM were shown to express TGF-β1 [96]. Whether the same holds true for the metastatic pediatric BM environment remains to be elucidated.

Furthermore, matrix metalloproteinase-9 (MMP-9) contributes to EMT by remodeling the extracellular matrix (ECM) and thereby facilitates invasion [97]. In head and neck squamous cell carcinoma, tumor cells have been found to instruct BM-MSCs to secrete MMP-9 in a three-dimensional spheroid system [98]. In NB, however, MMP-9 has only been shown to be present in the tumor-surrounding stroma, consisting of fibroblasts and (peri-)vascular cells [99], but not specifically to be derived from MSCs. Interestingly, MSCs might nevertheless contribute to the MMP-9 pool in the TME by inducing its expression in NB cells, as shown by stimulation of NB cell lines with conditioned medium from cultured MSCs [100]. Interestingly, MMP-9 was also found to be upregulated in high-risk NB tumors [99,101], indicating that this enzyme might play an important role in the dissemination process in NB (Fig. 1C).

Moreover, the reprogramming of adrenergic to mesenchymal NB cells was found to be mediated by a Notch feedforward loop [102,103]. Although the factors inducing this Notch signaling in NB remain to be unraveled, in vitro studies on acute myeloid leukemia (AML) suggest an involvement of MSCs: MSCs from AML patients expressed higher levels of Notch ligands and -receptors than MSCs from healthy donors and induced Notch signaling in AML cells in a coculture system [104].

### BM invasion

NB metastasizes to distinct secondary organs, preferentially the BM, which suggests that this invasion depends on interaction with resident cells and signaling factors. One prominent signaling axis involves CXCR4 and its ligand CXCL12: Early research showed that NB cells express CXCR4, which seems to play a critical role in metastasis to the BM [105,106] and that the level of CXCR4 expression is correlated with BM metastasis and poor clinical outcome [107]. Later, in vitro studies suggested that NB cells use the same CXCR4/CXCL12 axis for metastasis as HSPCs do for homing after stem cell transplantation [108] and that this process is supported by MSCs (Fig. 1D): Upon incubation with MSC-conditioned medium, NB cells showed increased migration and invasiveness, which was dependent on the CXCR4/CXCL12 axis [68,100,109].

Similarly, PC cells are also known to make use of the CXCR4/CXCL12 axis for BM metastasis [110,111]. Furthermore, circulating melanoma cells have been described to interact with perivascular MSCs through CXCR4/CXCL12 signaling and melanoma cell adhesion molecule (MCAM, CD146) in vivo, an interaction shown to be required for BM invasion [112]. Interestingly, recent study from our group with primary patient samples has determined CD146 to be one of the surface molecules that identifies an MSC subtype, which is specifically present in the NB metastatic BM and might have tumor-related functions [113].

Other studies proposed a role of CXCR5 and CXCR6 in migration of NB cells to the BM [114]. Invasion into the BM could furthermore be mediated by insulin-like growth factor 1 (IGF-1) receptors on NB cells and the high expression of IGF ligands in the bone, allowing NB cells to bind to BM-endothelial cells and migrate through the endothelium toward the IGF-1 pool in the BM environment [115] (Fig. 1D).

### Premetastatic niche

Since NB dissemination has a clear affinity for certain organs, including the BM, the idea of a favorable premetastatic niche (PMN) in the BM microenvironment comes to mind. The PMN concept is based on the idea that circulating tumor cells require a supportive niche at the secondary organ to establish metastases [116]. Although Paget described his “seed and soil” hypothesis about an interaction between tumor cells and their future sites of metastasis already in 1889 [117], the principle of a PMN was only confirmed many years later upon the discovery that melanoma-conditioned medium causes Lewis lung carcinoma cells to metastasize into typical melanoma metastatic sites instead of the lung [118]. The tumor cell secretome and EVs have been proposed as the cause for this distant effect [116], with organotropism being determined by the characteristic secretion profile of individual tumors [119].

PMN formation has been extensively studied in common tumors such as breast cancer, PC, and melanoma, and typical metastasis sites include lymph nodes, liver, bone, and brain [116]. As the majority of NB patients already present with BM metastasis at diagnosis, determining the role of the PMN in NB is difficult and thus not well understood. Nevertheless, understanding the potential role of factors and EVs secreted by NB primary tumors could be of key importance to prevent further metastasis and relapse. Since MSCs represent an important interaction partner of NB cells at the primary tumor site, BM-MSCs could also play a role as a distant messenger preparing the BM niche for metastatic invasion.

## MSCs at the BM Metastatic Niche of NB

As the primary site of some hematological malignancies and the main metastatic site of several solid tumors [120–122], the BM microenvironment is subject to intensive investigations in tumors such as multiple myeloma (MM), breast cancer, and PC. Yet, the interactions between NB cells and BM-MSCs are only starting to be investigated, with a few studies indicating a crosstalk of NB cells with BM-MSCs. Interestingly, our group recently demonstrated in primary NB patient samples that the number of MSCs is significantly increased in metastatic BM compared with NB-free BM, pointing toward a direct or indirect effect of NB cells on MSCs [113].

Crosstalk between tumor cells and their environment can occur in a direct manner through membrane protein interaction and integrin signaling or indirectly through cytokines, chemokines, growth factors, and EVs. Apart from their potential role in creating a PMN at the BM, tumor-derived EVs may also have tumor-supportive effects after the invasion of tumor cells into the BM [116].

A proteomic analysis of EVs derived from NB cell lines demonstrated the presence of proteins such as prominin-1, B7H3, basigin, and fibronectin on EVs, which are associated with cell survival and proliferation as well as chemoresistance, immune evasion, and ECM destruction [123–127]. Interestingly, the NB EV signature is suggested to be site- and stage-specific, as EVs secreted by BM-resident NB cells differ from those derived from primary and brain-metastasized NB cells [128], suggesting that they might fulfil distinct functions at their respective location.

**FIG. 2.**
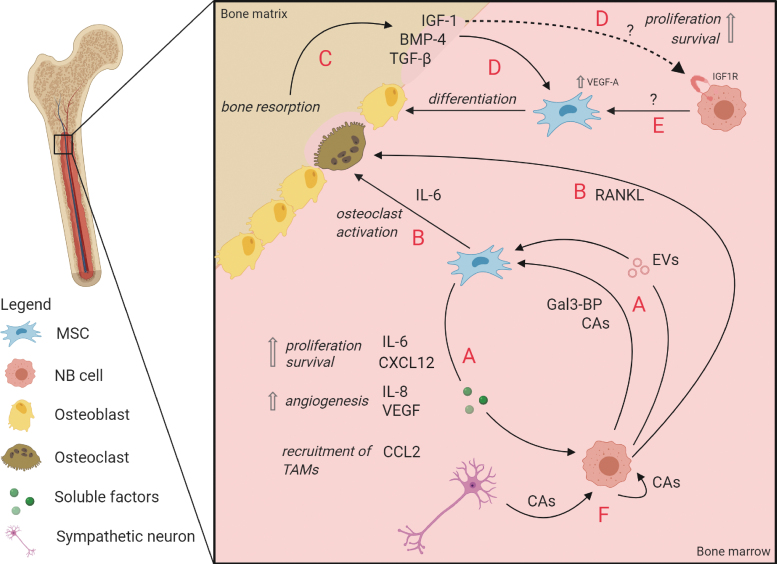
Interactions between MSCs and NB cells in the BM metastatic niche. **(A)** Several mediators, such as EVs [129], Gal-3BP [133,134], and potentially CAs [169,170] can influence MSCs to secrete tumor-supportive factors that increase NB cell proliferation and survival [109,135,151], promote angiogenesis [130,131] and recruit TAMs to the TME [132]. **(B)** MSCs, stimulated by Gal-3BP and other unknown NB-derived mediators, secrete IL-6, thereby increasing osteoclast differentiation and driving osteolysis [151]. NB cells secrete RANKL with a similar effect [150]. **(C)** Increased osteoclastic activity leads to additional bone resorption, releasing bone-derived growth factors (TGF-β, BMP-4, and IGF-1) into the marrow [152–154]. **(D)** BMP-4, IGF-1, and TGF-β increase osteoblastic differentiation of MSCs [152,153,155]. In addition, IGF-1 could potentially support NB cell survival and proliferation through interaction with IGF-1R (*dashed line*) [157]. **(E)** Unknown NB-derived factors drive differentiation of MSCs into osteoblasts through intrinsic VEGF-A signaling [153]. **(F)** It is hypothesized that CAs create a tumor-supportive environment [167]. NB cells might use this mechanism in both para- and autocrine ways to promote tumor progression. CAs, catecholamines; EVs, extracellular vesicles; Gal-3BP, Galectin-3 binding protein; RANKL, receptor activator of nuclear factor kappa-B ligand; TAMs, tumor-associated macrophages; TGF-β, transforming growth factor-β; TME, tumor microenvironment; VEGF, vascular endothelial growth factor. Color images are available online.

EVs derived from NB cell lines have been demonstrated to affect BM-MSCs: they stimulated the secretion of tumor-supportive cytokines and chemokines from BM-MSCs in vitro, most notably IL-6, IL-8/CXCL8, vascular endothelial growth factor (VEGF), and CCL2/MCP-1 [129] (Fig. 2A). While IL-8 and VEGF are known stimulators of angiogenesis [130,131], CCL2/MCP-1 has been demonstrated to promote the recruitment of anti-inflammatory tumor-associated macrophages [132]. Interestingly, IL-6 is a component frequently implicated in tumor-supporting pathways. In NB, its effect is believed to be controlled by a positive feedback loop: NB-derived Galectin-3 binding protein (Gal-3BP) activated the Ras/MEK/ERK pathway in MSCs in vitro, which in turn produced IL-6 [133,134] (Fig. 2A). In a STAT3/ERK1,2-dependent manner, IL-6 promoted proliferation and survival of tumor cells, protected them from drug-induced apoptosis in vitro and stimulated tumor growth in vivo [135]. Of note, these studies [133,135] used “BM stromal cells” that have not been confirmed to be MSCs based on surface marker expression or differentiation capacity. Treatment of NB cell lines with the chemotherapeutic agent sorafenib corroborates the aforementioned findings, as it blocked the IL-6-induced STAT3 phosphorylation and downstream signaling, inducing apoptosis and cell growth arrest of NB cells [136].

Similarly, IL-6/STAT3 signaling has been described in other tumors such as osteosarcoma [137], and targeting this axis has been proposed for ablating tumor-stroma crosstalk [138]. However, targeting IL-6 with receptor blocking antibodies alone seems inefficient, as several alternative pathways lead to STAT3 activation (an overview is given in Wendt et al. [139]).

In conclusion, secreted factors and the content of NB-derived EVs can stimulate the secretion of tumor-supportive factors from BM-MSCs and thereby contribute to communication with the TME to increase NB growth in the BM. Other components of NB-TME crosstalk and their molecular mechanisms remain to be elucidated before we can understand the complex interactions that sustain NB BM metastases.

### Bone homeostasis in the metastatic BM niche

The bone is a dynamic tissue subjected to constant remodeling by osteoclasts and osteoblasts, which resorb bone matrix and form new bone material, respectively [140]. Their activity is tightly regulated, resulting in a well-balanced equilibrium of bone homeostasis [141]. However, when tumor cells proliferate in the BM, this homeostasis is disturbed and can lead to osteolytic or osteoblastic lesions. While breast cancer and MM metastases are predominantly osteolytic, characterized by increased osteoclast activity and bone resorption, PC lesions are predominantly osteoblastic [142–146].

In these osteolytic tumors, osteoclastogenesis is activated by PTH-related protein (PTHrP) secretion by the tumor cell or by receptor activator of nuclear factor kappa-B ligand (RANKL) secreted by the tumor cell and/or BM-MSCs [147,148]. Clinically, NB BM metastases have been described to be predominantly of osteolytic nature, which has been confirmed by an increase of osteoclasts in histological examinations of NB bone lesions in a xenograft mouse model [149] and osteoclast activation through upregulation of PTHrP and RANKL in NB cells that were implanted into the femur of mice [150]. In another study, however, it was shown that various NB cell lines that induce osteolytic lesions in mice, did not secrete the osteoclast-activating factors themselves [151]. An alternative way of osteoclast activation through BM-MSC-derived IL6 was demonstrated in vitro in a coculture system of rat osteoclasts, BM-MSCs, and NB cell line CHLA-55. Only in the presence of BM-MSCs, an increased osteoclast activation was observed, which was dependent on IL6, secreted by BM-MSC solely upon contact with NB cells [151] (Fig. 2B).

The subsequent bone resorption does not only create space for tumor growth, but also leads to the release of growth factors such as TGF-β, bone morphogenetic factors (BMPs), and IGFs from the bone matrix (Fig. 2C), which in turn can increase osteoblastic differentiation [152–155] (Fig. 2D). Furthermore, IGFs have been shown to increase survival and proliferation in NB cells, PC cells, and MM cells in vitro, suggesting that IGF-1 released from the bone matrix in the proximity of metastatic tumor cells could also directly benefit tumor progression [156–158] (Fig. 2D).

In contrast to osteoclastic lesions, PC bone metastases are predominantly osteoblastic [159]. Furthermore, AML cells have been demonstrated to induce osteogenic differentiation of MSCs in vitro through BMP-Smad1/5 signaling [160]. Interestingly, several studies also investigated the involvement of osteoblasts in NB, but the results are contradicting. On the one hand, NB cells seemed to impede MSC differentiation into osteoblasts by secretion of Wnt-inhibitor Dkk1 in an in vitro model [161], a process that has likewise been described for osteolytic bone metastases of MM and breast cancer [162,163]. On the other hand, a study with murine BM-MSCs demonstrated that NB cells increased the in vitro differentiation of MSCs into osteoblasts by increasing the expression of intracellular VEGF-A [153] (Fig. 2E). This enhanced the effects of BMP-4, which is—next to Wnt- and Notch signaling—part of one of three pathways that control osteoblastogenesis [164]. Importantly, recent study from our group with ex vivo analyses of NB patient-derived material demonstrated BM-MSCs from metastatic NB patients to be more prone to differentiate toward osteoblasts compared to MSCs from patients without BM metastases [113].

In conclusion, the regulation of bone homeostasis in NB and the involvement of the BM stroma are complex and seem to implicate both osteolytic as well as osteoblastic processes. The latter represent “two extremes of a continuum” [165] and are thus coinciding events. Although bone metastases of most tumors present lesions that display both processes, they are termed “osteolytic” or “osteoblastic” based on the predominantly occurring process [159,166]. The benefit for the tumor is in both cases an increased availability of growth factors, either when being released from the bone matrix (in osteolytic lesions) or produced by an increased number of bone cells (in osteoblastic lesions) [158].

### Catecholamines in NB

Other important players in the metastatic BM environment are catecholamines, such as dopamine, epinephrine, norepinephrine, and their metabolites. In normal situations, catecholamines are primarily secreted in a circadian rhythm by sympathetic neurons and are involved in regulating activity and homing of HSPCs to the BM [167]. More specifically, secretion of norepinephrine by sympathetic neurons in the BM downregulates CXCL12 expression by stromal cells, resulting in HSPC release into the blood [168]. As a tumor originating from the neural crest, nearly all NB tumors secrete catecholamines and their metabolites, some of which are utilized as diagnostic markers [5]. Although their function in NB is unknown, they were found to promote tumor proliferation and metastasis in several other tumors [167]. This makes the NB metastatic niche particularly interesting and unique, as NB cells contribute to catecholamine production. Interestingly, MSCs from adipose tissue express various adrenergic receptors [169], and catecholamines were suggested to regulate MSC differentiation and migration (as reviewed by Hajifathali et al. [170]). Considering these findings, one could speculate that NB cells may utilize catecholamines to create a proliferative environment in an autocrine or paracrine (to MSCs) manner (Fig. 2F), or to assist in the creation of space for the tumor within the BM niche by expelling HSPCs [171].

## Therapy Resistance and Dormancy

Since >30% of NB patients relapse after complete remission [1], it is essential to understand therapy resistance and MRD in the BM. Whereas most macroscopic tumor lesions respond to therapy, are resected, and become undetectable, some cells may evade therapy, persist, and remain undetected [6]. Although the majority of studies focuses specifically on resistance to chemotherapy, there are also efforts to elucidate resistance to other therapeutic approaches such as immunotherapy (discussed hereunder).

Chemoresistance can arise intrinsically (acquired chemoresistance) or be mediated by cells in the TME [environment-mediated drug resistance (EMDR)] [172]. The latter can be facilitated by soluble factors and EVs from the TME as well as by cell adhesion to the ECM or stromal cells [17]. Furthermore, dormancy of tumor cells enables them to escape treatment, since chemotherapeutic agents often target fast-dividing cells in a nonspecific way [173]. Dormancy on the cellular level is defined by mechanisms that induce cellular quiescence, that is, a reversible nonproliferative state [15,174].

A contribution of MSCs to chemoresistance and dormancy has been demonstrated in several cancer types. In breast cancer, for example, MSCs have been described to promote chemoresistance and induce tumor dormancy by secreting cell cycle-inhibitory miRNAs and creating a tumor-protective niche [175,176]. Breast cancer cells were also shown to enter a dormant state in vitro after cannibalizing BM-MSCs, after which they acquired a senescence-associated secretome [177]. In bone metastatic PC, BMP-7, which normally regulates HSC dormancy, was secreted by BM-MSCs and induced a reversible senescence-like state in the tumor cells by inhibiting EMT [178].

In the BM metastatic setting of NB little is known about the processes leading to dormancy and therapy resistance. However, in in vitro settings and in the in vivo environment of the primary tumor the contribution of MSCs to therapy resistance has been investigated. Chemoresistance in NB was shown to involve MSC-mediated STAT3 signaling in in vitro experiments: NB cells cocultured with patient-derived BM-MSCs were protected from etoposide-induced apoptosis [73,179,180]. The results suggested Sphingosine-1-phosphate receptor 1 (S1PR1) to play a role in the activation of STAT3 signaling in NB cells and showed that antiapoptotic proteins Bcl2 and survivin are involved in the STAT3-related chemoresistance mechanism [179,180]. Consistently, knockdown or inhibition of S1PR1 abrogated the STAT3-mediated chemoresistance [179]. These results are corroborated by in vivo studies: inhibition of STAT3 with AZD9150 increased sensitivity of NB to cisplatin, as seen by decreased tumor growth (64%) and significantly prolonged survival of mice [181]. Furthermore, combined inhibition of STAT3 (by ruxolitinib) and ERK1/2 (by trametinib) sensitized NB cells to etoposide and led to decreased tumor size and prolonged survival of mice [73].

Resistance to anti-GD2-immunotherapy was mediated by BM-MSCs in an in vivo study: BM-MSCs isolated from NB patients, co-injected with monocytes into the renal capsule of mice, protected NB cells from toxicity induced by dinutuximab (an anti-GD2 antibody) and activated natural killer cells (aNKC) [182]. Whether these BM-MSCs were isolated from BM with metastases, where they might have been manipulated by tumor cells to become protective, was not addressed in this study. Addition of an anti-CD105 (Endoglin) antibody restored the efficiency of the aNKC/dinutuximab treatment. Since the anti-CD105 antibody eliminates not only MSCs but also monocytes and endothelial cells, the protective effect cannot be attributed solely to MSCs here. Based on analyses of conditioned medium from cocultures of MSCs, monocytes and NB cells, TGF-β1 was proposed to be a major contributor to MSC-/monocyte-induced protection from aNKC/dinutuximab treatment [182]. Corroborating this hypothesis, another study reported inhibition of TGF-βR1 with galunisertib to restore antitumor activity of the aNKC/dinutuximab combination treatment in vitro and in vivo [183].

These studies provide intriguing evidence for the contribution of MSCs and some molecular mechanisms of therapy resistance. Further research into factors and signaling pathways involved in MSC-mediated therapy resistance in the BM is needed to advance our understanding of the mechanisms that underlie NB relapse.

## Clinical Perspective

### MSCs as cellular therapy

Because of their multipotent nature, MSCs are often used in regenerative medicine and in addition to treatment for a variety of nonmalignant diseases [184,185]. Although a range of studies show tumor-supportive properties of MSCs, a potential clinical use of MSCs in tumor therapy is being investigated. The safety of such application must, therefore, be taken into account and be treated with caution [186].

One property of interest is their hematopoietic supportive function to promote recovery of the hematopoietic system after myeloablative cancer therapy and stem cell transplantation. MSC co-transplantation can be used to support the nesting of HSPCs in the BM hematopoietic niche, to reduce the inflammation of damaged tissue and thus to sustain an overall functional BM niche [187]. Although the benefits of MSC co-transplantation to enhance engraftment in allogeneic HSC transplantation and to prevent graft-versus-host disease have been studied extensively [188], its use in the autologous context, as common in NB, remains largely unexplored [189,190].

The second MSC property with a potential clinical benefit is their tumor-tropism to selectively deliver anticancer agents to tumors. In NB tumors, a few possible agents have been tested in vitro and in vivo, including TNF-related apoptosis-inducing ligand (TRAIL) [191], interferon-gamma (IFN-γ) [192], IFN-β [193], and the neuronal differentiation-associated microRNA miR-124 [194]. Furthermore, the use of oncolytic virus-infected MSC products has been tested in vivo [195] and showed only small side effects in NB therapy in a phase I/II clinical trial [196].

However, there is evidence that indicates rapid clearance of ex vivo expanded MSCs after systemic administration [197], questioning the ability of MSCs to migrate to their target tissue in NB therapy. Utilizing EVs as a delivery vehicle instead could present a remedy for this limitation: The successful targeting to tumors and effectiveness of EVs loaded with oncolytic virus or chemotherapeutic agents was demonstrated in a mouse model of lung cancer [198] and human BM-MSC-derived EVs resulted in a therapeutic effect in a graft-versus-host disease model [199].

In addition, MSCs might exert an adverse effect on tumor progression, which could diminish the intended benefit of these therapies. The multitude of possible applications of MSCs in cellular therapy stress the need to further investigate the role of MSC-NB crosstalk to ensure their safe use in the clinical setting.

### Therapy targeting MSC-NB crosstalk

In addition to the aforementioned approaches that exploit the beneficiary functions of MSCs, there are other endeavors that try to directly target MSCs and the TME they sustain to ablate their tumor-supportive effect, especially their therapy-protective functions. Therapy resistance of tumor cells in BM remains a major obstacle for curing NB [13,17]. Treatment should, therefore, aim to address EMDR effectively and increase chemotherapy efficiency, for example, by mobilizing NBCs from their protective environment in the BM niche. Secondly, targeting the BM more specifically would aid in reducing the chemotherapeutic load for patients. Strategies for achieving the latter have extensively been reviewed by Mu et al. [200]. The following section summarizes existing knowledge about intriguing new ways of targeting NB cells and MSCs and their interaction with the TME to overcome chemoresistance and eliminate MRD.

#### Targeting NB–MSC interactions in the TME

The CXCR4/CXCL12 axis is an interesting candidate for targeting the BM TME because of its important role in NB metastasis and progression and the contribution of MSCs to this signaling axis, as mentioned earlier in this review. The feasibility is supported by two studies that show reduced primary NB growth in vivo, one using virally delivered and the other systemically injected CXCR4 antagonists [201,202]. In addition, an inhibitory effect on NB proliferation and metastasis, partly due to reduced CXCR4 expression, is observed upon use of isatin, an endogenous indole found in plants and humans [203]. Finally, enhanced CXCR4 expression was found in cisplatin-resistant tumors, and inhibition of CXCR4 expression on NB cells with the VEGFR-inhibitor vandetanib restored cisplatin sensitivity in mice [204].

Directly eliminating BM-MSCs is another approach to abolish their tumor-supportive effects. One of the established targets on MSCs is the transmembrane receptor CD105 (also targeting monocyte and endothelial cells), to which antibody-dependent cellular cytotoxicity by anti-CD105 antibodies can be directed. An in vivo study in mice showed that resistance to anti-GD2 immunotherapy of NB conferred by MSCs and/or monocytes can be overcome by eliminating these cells with anti-CD105 antibodies [182].

Another approach targeting the BM niche and reducing the burden of osteolytic lesions in metastatic NB is to interfere with RANK/RANKL signaling. Endogenously, the RANKL decoy receptor osteoprotegerin (OPG) inhibits osteoclast activation [140,166]. A phase III clinical trial for treatment of osteolytic lesions in MM patients, showed the efficacy and safety of RANKL inhibitor denosumab, which mimics the endogenous OPG effects [205]. Its application in NB has not been investigated yet, but could prove beneficial to prevent osteolysis and the concomitant effects on tumor progression and to provide supportive care for bone disease in NB.

#### Mobilizing NB cells out of the protective BM niche

Although not directly MSC related, the mobilization of sequestered NB cells out of the protective environment of the BM niche is a valuable approach for improving therapy success. Tumor cells thereby lose their (indirect) contact with MSCs and other cells in the TME and become more accessible for tumor-targeting drugs. Since mobilization could introduce the risk of new metastases, this should be done with great caution and accompanied by a consecutive chemotherapy course. Nevertheless, there are some studies that support this idea, for example, by targeting CXCR4 or adhesion molecules such as integrins.

In a breast cancer xenograft model, the CXCR4-specific inhibitor AMD3100 [206] successfully mobilized dormant tumor cells out of the perisinusoidal niche of the BM, as demonstrated with real-time in vivo imaging [207]. Similarly, AMD3100 diminished adhesion of MM cells to BM stromal cells (identity not further clarified) in vitro and promoted mobilization of tumor cells into the circulation in vivo, subsequently sensitizing them to bortezomib [208]. However, AMD3100 presents a nonspecific way of targeting tumor cells in the BM, as it is also used for mobilizing HSPCs from the BM before stem cell transplantation [209]. All AMD3100-effects must, therefore, be considered during its application, but it might be beneficial in sensitization of NB cells to therapy and specific targeting of dormant NB cells to lower the risk for relapse.

A more precise target for blocking NB cell adhesion could be tumor-specific integrins, which regulate tumor migration, invasion, and adhesion to the ECM, and whose high expression is associated with increased metastasis [210]. For instance, integrin subunits α3β1were previously reported to be upregulated in NB cells exposed to conditioned medium from BM-MSCs, concomitant with increased invasiveness, which implies a functional role for NB metastasis and attachment [100]. Furthermore, combined inhibition of αVβ3 and αVβ5 integrins reduced NB cell attachment to the culturing surface and thereby increased the cytotoxic effects of an anti-GD2 antibody in vitro [211]. Overall, targeting adhesion by blocking integrins and/or CXCR4 might pose an exciting new way to promote mobilization of NB cells out of the BM and sensitize them to treatment.

## Open Questions and Outlook

Although an increasing body of evidence suggests a tumor-supportive role of MSCs in various tumor types, more *in/*ex vivo research is necessary in the context of NB to confirm previous findings and extend our knowledge regarding the role of MSCs in EMT, chemoresistance, and dormancy.

**FIG. 3.**
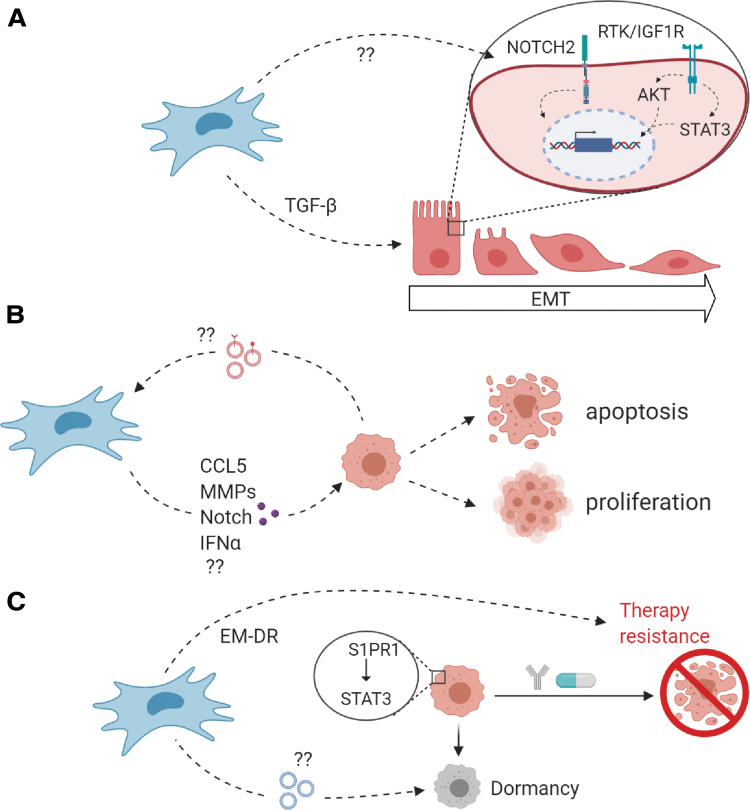
Open questions regarding NB–MSC interactions. **(A)** Metastasis is dependent on the cell's capacity to migrate to distant sites. Whether MSCs are a source of TGF-β and MMP-9 or activate Notch signaling in NB cells, all of which are known to be involved in EMT and invasion in NB [93,100,102], remains to be elucidated in NB. Furthermore, the PI3K/AKT pathway and STAT3 signaling have been implicated to contribute to EMT [94,95]. **(B)** Additional signaling between NB cells and MSCs through cytokines, chemokines, and growth factors (*purple*) might contribute to tumor proliferation and -survival. CCL5 [69], MMPs [98], and Notch signaling [213] have been described to contribute to cancer cell motility, invasion, and differentiation into CAFs. MSC-derived IFNα, in contrast, was suggested to inhibit proliferation of cancer cells [212]. Furthermore, the cargo of exosomes derived from metastatic NB cells (*red*) and the signaling it induces in MSCs is an interesting field of research [123]. **(C)** To prevent EMDR and induction of dormancy through MSCs, the signaling components from MSCs contributing to these processes need to be studied in detail. It has been described that MSCs induce expression of S1PR1 in NB cells, which protected NB cells from drug-induced apoptosis through the JAK-STAT3 signaling pathway [179]. In breast cancer, miRNA-loaded exosomes promoted quiescence in tumor cells [175,176]. CAF, cancer-associated fibroblast; EMDR, environment-mediated drug resistance; IFNα, interferon α; RTK, receptor tyrosine kinases. Color images are available online.

It remains unclear if and to what extent MSCs promote EMT in NB cells, for example. Are they involved in the Notch signaling that induces the switch between adrenergic and mesenchymal NB cells? Could MSCs in the pediatric BM be a source of TGF-β that evokes EMT characteristics in NB cells (Fig. 3A)? What role do EVs and other pro- or antitumorigenic components (such as CCL5 [69], MMPs [98], IFNα [212], and Notch signaling [213]) play in NB progression in the BM (Fig. 3B)? And by what mechanisms do MSCs contribute to EMDR and dormancy in NB cells (Fig. 3C)? These and more questions need to be answered to find out how to harness the MSC-NB crosstalk to our advantage. The complexity of signaling and crosstalk in the TME, which can differ depending on tumor localization and experimental design as well as on the well-known NB heterogeneity, have to be considered when interpreting results.

The fundamental knowledge of molecular mechanisms is imperative for designing new treatment options that target the tumor and its microenvironment in a more effective and specific way and thereby avoid unfavorable side effects. Furthermore, the development of targeted drug delivery to the BM is crucial for advancing the progress in curing NB.
